# Composition and In Vitro Activity–Cytotoxicity Profiles of *Vespa basalis* Venom: Combined Proteomic, Metabolomic, and Molecular-Weight-Fractionated Analyses

**DOI:** 10.3390/toxins18090394

**Published:** 2026-09-12

**Authors:** Yong-Hua Wu, Zheng-Xu Zhong, Yan Li, Jing-An Wang, Zheng-Wen Ou, Hou-Jin Li, Wen-Jian Lan

**Affiliations:** 1 State Key Laboratory of Anti-Infective Drug Discovery and Development, School of Pharmaceutical Sciences, Sun Yat-sen University, Guangzhou 510006, China; wuyh276@mail2.sysu.edu.cn (Y.-H.W.); zhongzhx25@mail2.sysu.edu.cn (Z.-X.Z.); liyan526@mail2.sysu.edu.cn (Y.L.); 2 Production and Research Base for Wasp Deinsectization, Guangdong Huxin Biotechnology Co., Ltd., Jiangmen 529245, China; wangjingan@hufenghuang.com (J.-A.W.); 1ozw@163.com (Z.-W.O.); 3 School of Chemistry, Sun Yat-sen University, Guangzhou 510006, China

**Keywords:** *Vespa basalis* venom, proteomic profiling, metabolomic profiling, molecular-weight fractionation, LPS-induced nitric oxide, tumor-cell viability, THLE-2 cytotoxicity

## Abstract

*Vespa basalis* Smith venom (VBsV) causes painful and potentially severe systemic reactions, yet the relationships among its composition, bioactivity, and cytotoxicity remain poorly understood. Proteomic and metabolomic profiling was combined with ultrafiltration and gel filtration to characterize VBsV and obtain >3 kDa, operationally defined 700–3000 Da, and <700 Da fractions. The effect on LPS-induced NO production was evaluated by nitric oxide (NO) release in LPS-stimulated RAW264.7 cells, and cytotoxicity was assessed in tumor and THLE-2 liver cells. Five venom allergen proteins were identified, including two phospholipase A1 isoforms, hyaluronidase A, antigen 5, and venom dipeptidyl peptidase IV. Targeted profiling also detected several neurotransmitters and energy metabolites, including 5-hydroxytryptamine, L-glutamate, histamine, γ-aminobutyric acid, inosine, and adenosine. The 700–3000 Da fraction exhibited the strongest NO-suppressive and tumor-cell-viability effects among the tested fractions. The 700–3000 Da fraction suppressed NO release (IC_50_, approximately 23 μg/mL) and reduced tumor-cell viability, while minimally affecting THLE-2 cells at 10–80 μg/mL; by contrast, the >3 kDa fraction showed greater THLE-2 cytotoxicity. These findings identify the 700–3000 Da fraction as a priority for direct compositional characterization and isolation of the responsible molecules.

## 1. Introduction

The venoms of wasps and other hymenopteran insects represent a typical double-edged sword. On the one hand, venom serves as a potent biochemical weapon for defense against predators, and envenomation commonly causes intense local pain, severe edema, systemic allergic reactions, and, in extreme cases, fatal multiple-organ failure [1]. On the other hand, through a long evolutionary process, wasp venom has become a rich reservoir of naturally occurring bioactive molecules [2]. In recent years, several Vespa venoms and venom-derived peptides have been reported to possess anti-inflammatory, antimicrobial, and tumor-cell-inhibitory activities. In particular, mastoparan and related peptides have shown pronounced inhibitory effects against certain hematological tumor cells and melanoma cells [3,4,5,6,7,8]. However, venom is highly complex and comprises macromolecular enzymes, such as phospholipases and hyaluronidases, peptides such as mastoparans, and low-molecular-weight metabolites, including biogenic amines [3,9,10]. Although this compositional complexity underlies the diverse biological activities of venom, it also gives rise to systemic toxicities that are difficult to avoid and substantially restrict its direct clinical translation [3].

*Vespa basalis* Smith is regarded as one of the most aggressive and venomous hornet species in Asia [11]. Because its stings can cause severe pain, local tissue injury, and serious systemic reactions, early studies mainly focused on its toxicological mechanisms [12,13,14,15]. These investigations led to the isolation of phospholipase A1 (PLA1), which exhibits hemolytic and lethal activities [1]; mastoparan B, which induces local edema and cardiovascular depression [12,16,17,18]; and HP-series chemotactic peptides, such as HP-1 and HP-2, which display hemolytic activity [19]. Nevertheless, previous studies have largely focused on individual toxins and their acute toxicological effects, and the overall chemical composition and potential pharmacological activities of *V. basalis* venom remain insufficiently characterized. On the one hand, a systematic analysis of its small-molecule metabolites, bioactive peptides, and potential allergenic proteins within a unified analytical framework is still lacking. On the other hand, the distribution of target pharmacological activities and cytotoxicity risks among fractions of different molecular weights remains unclear. Although several vespa venoms have been reported to exert anti-inflammatory and tumor-cell-inhibitory effects in vitro, crude venom is a complex mixture of neuroactive small molecules, membrane-active peptides, and macromolecular toxic or allergenic proteins. Its apparent pharmacological effects may therefore arise from the combined actions of multiple classes of constituents. Activity-guided fractionation is consequently required to characterize the composition, target activity, and normal-cell cytotoxicity of different molecular-weight fractions and to identify a priority range with a relatively favorable in vitro activity–cytotoxicity balance.

Accordingly, this study aimed to systematically evaluate the bioactivities of *V. basalis* Smith venom (VBsV) and its different fractions in inflammation- and tumor-related models by combining proteomic and metabolomic profiling with an activity-guided fractionation strategy. Proteomic and metabolomic approaches were used to characterize the proteins, peptides, and low-molecular-weight constituents of VBsV. Crude venom was then operationally separated by ultrafiltration and gel filtration into a >3 kDa fraction, a 700–3000 Da fraction, and a <700 Da fraction. The effects of crude venom and the different fractions on LPS-induced NO production, cytotoxicity against tumor cells, and THLE-2 cell viability were subsequently compared. This study sought to demonstrate relationships among the composition, molecular-weight distribution, and in vitro bioactivities of VBsV and to provide new data for the analysis of the composition and activity of natural toxins of vespa venom.

## 2. Results

### 2.1. Identification of Characteristic Proteins in VBsV

**Figure 1:**
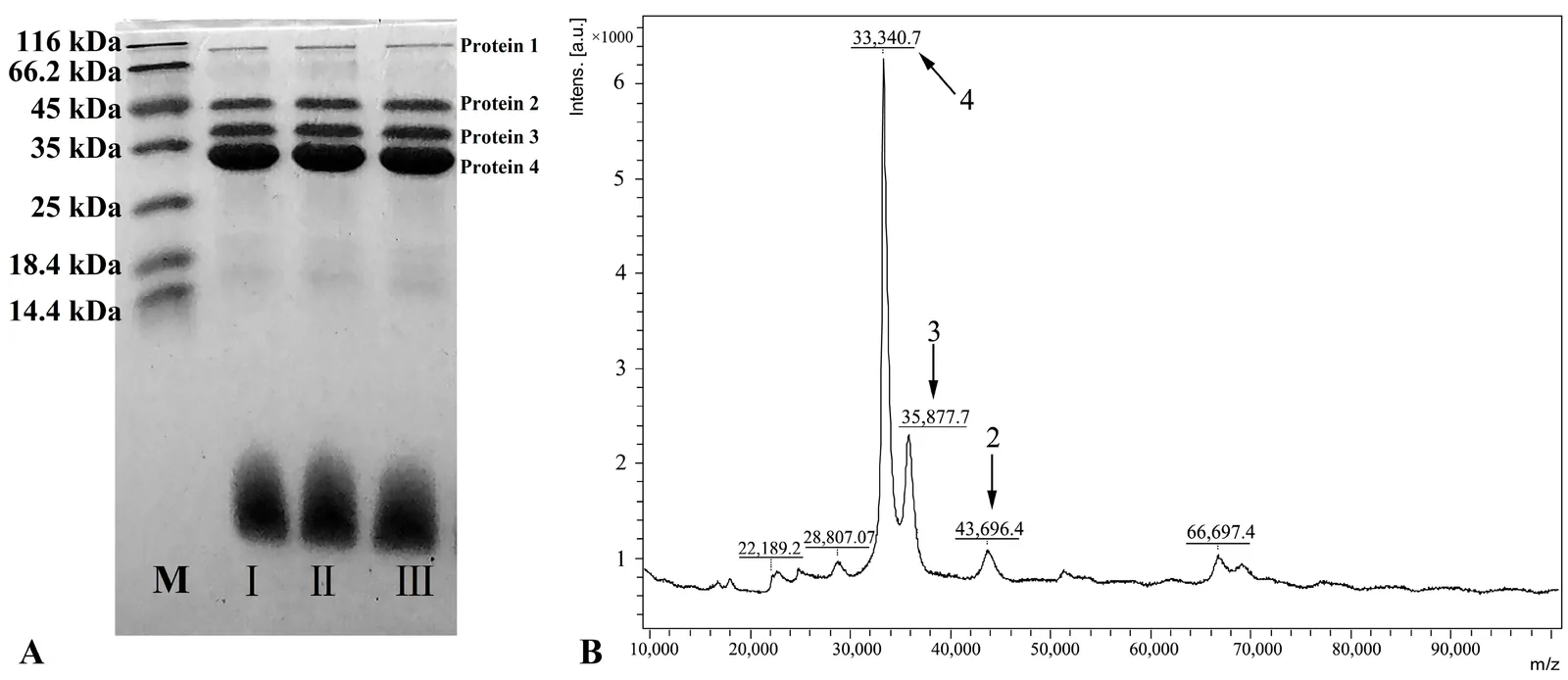
SDS-PAGE electrophoresis (**A**) and MALDI-TOF-MS mass spectrometry analysis (**B**) of *Vespa basalis* Smith venom (VBsV). In (**A**): I, II, and III represent three independently prepared batches of VBsV samples; M is the protein molecular-weight marker. In (**B**): peaks 2–4 correspond to the mass spectrometry signals of matched proteins 2–4, respectively.

To comprehensively characterize the macromolecular composition of VBsV, SDS-PAGE, MALDI-TOF-MS, and LC-MS/MS sequencing were combined for integrated identification. SDS-PAGE revealed four major protein bands in VBsV within the range of 25–116 kDa, which were designated proteins 1–4 (Figure 1A). Based on the molecular masses measured by MALDI-TOF-MS, proteins 2–4 had molecular masses of 43.7, 35.9, and 33.3 kDa, respectively (Figure 1B). Protein 1 exhibited an apparent molecular weight of approximately 80 kDa in SDS-PAGE electrophoresis, but no significant signal peak corresponding to its migration position was detected in the MALDI-TOF-MS spectrum. The 66.7 kDa peak is tentatively considered to be a dimer of protein 4.

In the global proteomic profiling of VBsV, protein 4 was identified as phospholipase A1 (PLA1), whereas proteins 1–3 were separately analyzed by in-gel tryptic digestion followed by LC–MS/MS and identified as venom dipeptidyl peptidase IV (DPP-IV; predicted molecular mass, 88.4 kDa), hyaluronidase A, and a PLA1 isoform, respectively (Supplementary Material Figure S1A–D). Global proteomic analysis also identified allergen 5, whose predicted molecular mass was consistent with a characteristic MALDI–TOF-MS peak at approximately 22 kDa (Supplementary Material Figure S2A). In addition to these macromolecular proteins, a mastoparan-like peptide 12a was identified with high confidence by LC–MS/MS, with 78.5% sequence coverage and six peptide-spectrum matches (PSMs) (Supplementary Material Figure S2B).

### 2.2. Analysis of the Characteristic Chemical Composition of VBsV

To characterize the chemical profiles of small-molecule compounds in VBsV, this study systematically analyzed three independent batches of venom samples using untargeted LC-MS/MS. The superimposed total ion current (TIC) chromatograms showed highly consistent retention times and overall ion intensity patterns for major peaks across the same group of samples (Supplementary Material Figure S3A,B). The overlaid TIC profiles showed similar retention-time patterns and major-ion profiles among the three independent batches, supporting batch-to-batch comparability.

**Figure 2:**
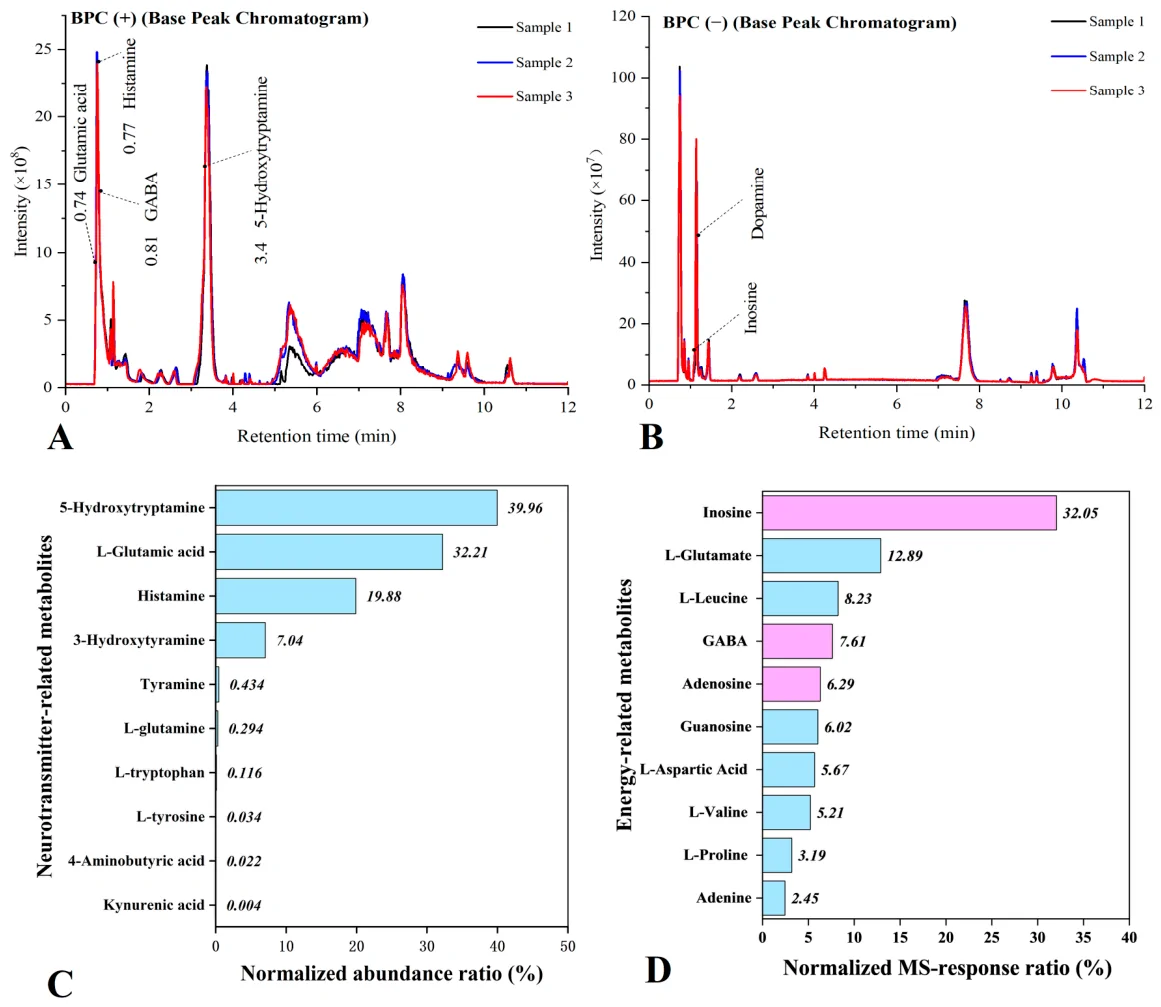
Characteristic chemical composition analysis of VBsV. (**A**) Base peak chromatogram (BPC) from untargeted metabolomics in positive ion mode; (**B**) base peak chromatogram (BPC) from untargeted metabolomics in negative ion mode; (**C**) top 10 most abundant neurotransmitters detected in targeted neurotransmitter analysis; (**D**) top 10 most abundant energy metabolites detected in pseudo-targeted energy metabolite analysis.

Untargeted LC-MS/MS data processing and database matching yielded 720 putatively annotated features (Supplementary Data S1). Chemical-class annotation showed that these candidates were mainly assigned to organoheterocyclic compounds, lipids and lipid-like molecules, organic acids and derivatives, nucleoside/nucleotide analogues, and amino acids, peptides, and related compounds, indicating that the reconstituted venom sample possessed a complex small-molecule chemical fingerprint. Notably, several candidate neuroactive compounds were observed in the untargeted dataset, including histamine, serotonin, glutamate, dopamine, and kynurenic acid. Several candidates associated with energy metabolism were also detected, including inosine, γ-aminobutyric acid, and adenosine derivatives. These findings suggest that the small-molecule composition of VBsV may comprise a characteristic profile formed jointly by neuroactive molecules, purine/nucleoside metabolites, amino-acid metabolites, and organic acids (Figure 2A,B; BPCs).

Because the untargeted annotations were primarily based on database matching, targeted LC-MS/MS analyses were further used for the semiquantitative characterization of key neurotransmitters and energy metabolism. Fifteen neurotransmitter-related small molecules were detected (Supplementary Material, Table S1). The four neurotransmitter-related molecules with the highest relative abundances were serotonin (5-hydroxytryptamine, 5-HT), L-glutamate, histamine, and dopamine (Figure 2C). These molecules are closely associated with neuronal excitation, pain perception, vascular responses, and inflammatory regulation. Targeted analysis of energy metabolites provided relative-abundance information for 56 small molecules associated with energy metabolism (Supplementary Material, Table S2), among which inosine, adenosine, and γ-aminobutyric acid (GABA) generated among the highest MS responses within the analyzed panel (Figure 2D). Previous studies have associated these molecules with anti-inflammatory responses and negative-feedback regulation of immunity. Collectively, these results indicate that the small-molecule composition of VBsV contains both molecules related to neuronal excitation, nociception, and inflammation and molecules with potential anti-inflammatory or immunomodulatory activities, providing a basis for further understanding its chemical characteristics and potential biological functions. Since the targeted analysis of neurotransmitters and energy metabolites was conducted based on a single representative batch, the results are limited to compound identification and relative abundance comparison.

### 2.3. In Vitro Effects of VBsV on LPS-Induced NO Production and Cell Viability

**Figure 3:**
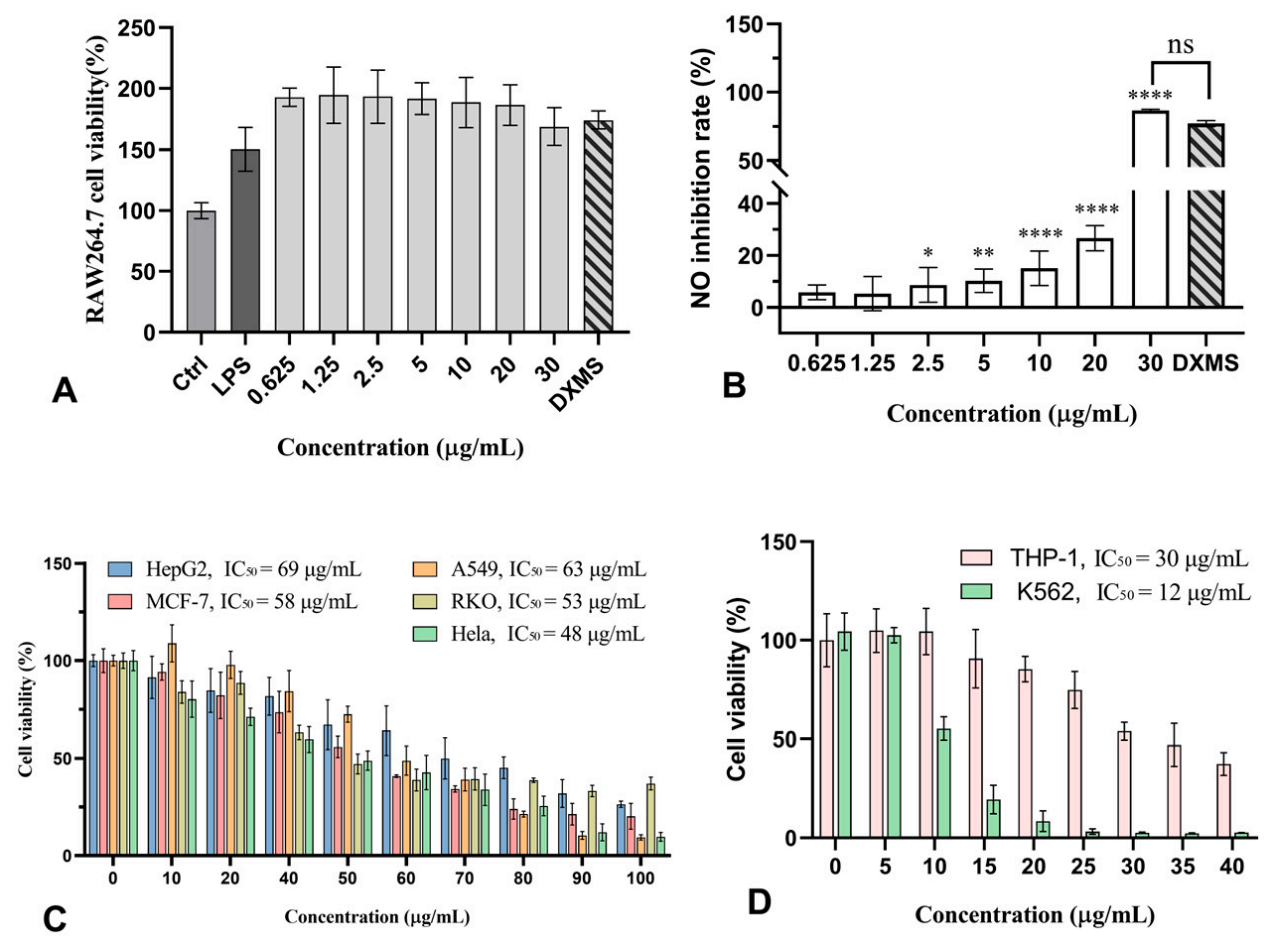
Effects of VBsV on LPS-induced NO production and cell viability after 24 h. (**A**) Relative CCK-8 signal in RAW264.7 cells treated in parallel with the NO assay; (**B**) NO inhibition rate in supernatants from LPS-stimulated RAW264.7 cells; (**C**) CCK-8 viability responses of solid-tumor-derived cell lines; (**D**) CCK-8 viability responses of hematological tumor-derived cell lines. Dexamethasone (DXMS, 10 μg/mL) served as the positive control for the NO assay. For (**B**), comparisons were made with the LPS model group; * *p* < 0.05, ** *p* < 0.01, **** *p* < 0.0001, and ns, not significant. Data are mean ± SD from three independent experiments, each with six technical replicate wells.

In the LPS-stimulated RAW264.7 macrophage inflammation model, none of the tested concentrations of VBsV caused significant cytotoxicity (Figure 3A), ruling out false positives caused by cytotoxicity. VBsV inhibited nitric oxide (NO) release in a concentration-dependent manner, with significant inhibition observed at 2.5 μg/mL (*p* < 0.05). The half-maximal inhibitory concentration (IC_50_) was 22 μg/mL, and at 30 μg/mL, the inhibitory effect was comparable to that of 10 μg/mL dexamethasone, a classical glucocorticoid anti-inflammatory drug (Figure 3B).

The broad-spectrum tumor-cell cytotoxicity of VBsV was evaluated using the CCK-8 assay. Across the five solid-tumor-derived cell lines, the IC_50_ values ranged from 48 to 69 μg/mL (HepG2, 69; MCF-7, 58; A549, 63; RKO, 53; and HeLa, 48 μg/mL), indicating a moderate inhibitory effect (Figure 3C). In contrast, hematological tumor cells were highly sensitive to VBsV. The IC_50_ against K562 leukemia cells was as low as 12 μg/mL, and VBsV also exerted a strong cytotoxic effect on THP-1 cells, with an IC_50_ of 30 μg/mL (Figure 3D).

### 2.4. Effects of VBsV Molecular-Weight Fractions on LPS-Induced NO Production and Tumor-Cell Viability

**Figure 4:**
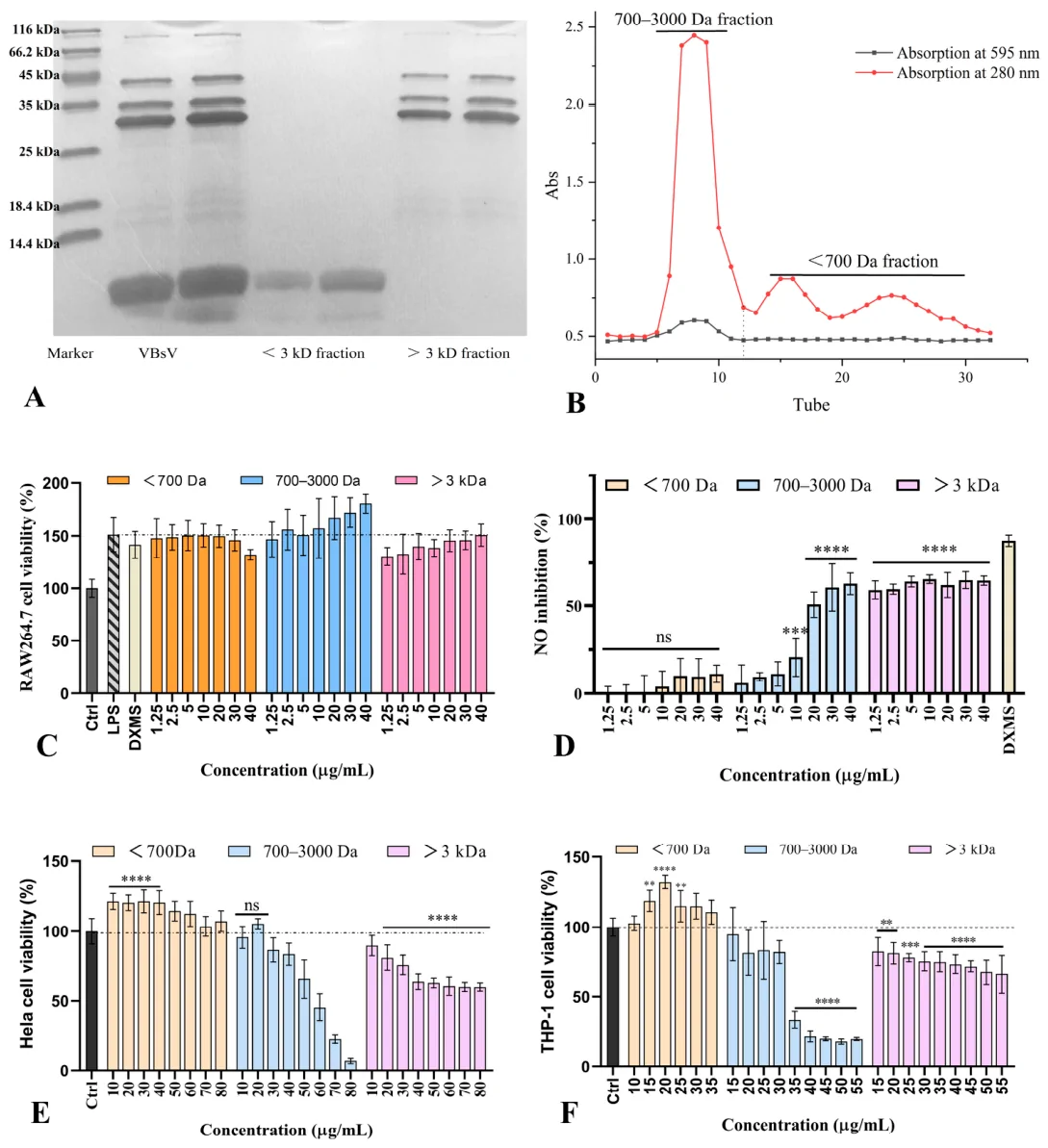
Fractionation of VBsV and comparison of concentration-normalized cellular effects. (**A**) SDS-PAGE analysis of the >3 kDa and <3 kDa fractions after ultrafiltration; (**B**) Sephadex G-10 elution profile used to operationally collect the 700–3000 Da and <700 Da fractions; (**C**) relative CCK-8 signal in RAW264.7 cells treated in parallel with the NO assay; (**D**) inhibition of LPS-induced NO production; (**E**) CCK-8 viability response of HeLa cells; (**F**) CCK-8 viability response of THP-1 cells. For (**D**), comparisons were made with the LPS model group; for (**E**,**F**), comparisons were made with the untreated control. ** *p* < 0.01, *** *p* < 0.001, **** *p* < 0.0001, and ns, not significant. Data are mean ± SD from three independent experiments, each with six technical replicate wells.

To compare concentration-normalized effects among molecular-weight fractions, VBsV was separated by ultrafiltration combined with Sephadex G-10 chromatography into three fractions: a <700 Da fraction, a 700–3000 Da fraction, and a >3 kDa fraction. Gel electrophoresis results confirmed effective separation of components >3 kDa and <3 kDa (Figure 4A); the elution profile from Sephadex G-10 chromatography, along with MALDI-TOF MS analysis of the operationally defined 700–3000 Da fraction (Figure 4B, Supplementary Material Figure S4), indicated successful separation, yielding fractions in the 700–3000 Da range and <700 Da. RAW264.7 cells, HeLa cells, and THP-1 cells were used as inflammation, solid-tumor, and hematological-tumor models, respectively, for systematic bioactivity evaluation. The fractions displayed markedly distinct biological-activity profiles.

In the LPS-induced NO production assay, none of the tested concentrations of the fractions showed significant cytotoxicity, ruling out false-positive results due to cytotoxicity. The results indicate that the <700 Da fraction did not significantly inhibit NO production at concentrations of 1.25–40 μg/mL, and its inhibition rate remained low. In contrast, the 700–3000 Da fraction produced a clear concentration-dependent inhibition of NO production, reaching relatively high levels at 10–40 μg/mL (*p* < 0.0001), with an IC_50_ of 23 μg/mL (Figure 4C,D). The >3 kDa fraction produced pronounced NO inhibition even at the lowest concentration tested (1.25 μg/mL), with an inhibition rate of approximately 60%. Further increases in concentration to 40 μg/mL did not substantially enhance the effect, which remained relatively stable overall (Figure 4C,D). These results indicate that the 700–3000 Da fraction exhibited a typical concentration-dependent NO inhibition response, whereas the >3 kDa fraction showed a pronounced response at low concentrations.

In the tumor-cell cytotoxicity assay, the <700 Da fraction showed no significant cytotoxicity toward either HeLa or THP-1 cells at 10–80 μg/mL.An increase in the CCK-8 viability signal was observed at some concentrations; however, its biological basis remains unknown. In contrast, both the 700–3000 Da and >3 kDa fractions exerted cytotoxic effects to varying degrees. Increasing concentrations of the 700–3000 Da fraction progressively reduced the viability of HeLa and THP-1 cells, with IC_50_ values of 56 μg/mL for HeLa cells and 33 μg/mL for THP-1 cells. The >3 kDa fraction exerted pronounced reductions in tumor-cell CCK-8 viability at relatively low concentrations, namely 20 μg/mL in HeLa cells and 15 μg/mL in THP-1 cells. However, the activity changed little as the concentration increased, and the maximum inhibition rates in both cell lines approached approximately 40%, suggesting that the response reached a plateau within the tested concentration range.

### 2.5. Effects of VBsV and Its Molecular-Weight Fractions on THLE-2 Cell Viability

**Figure 5:**
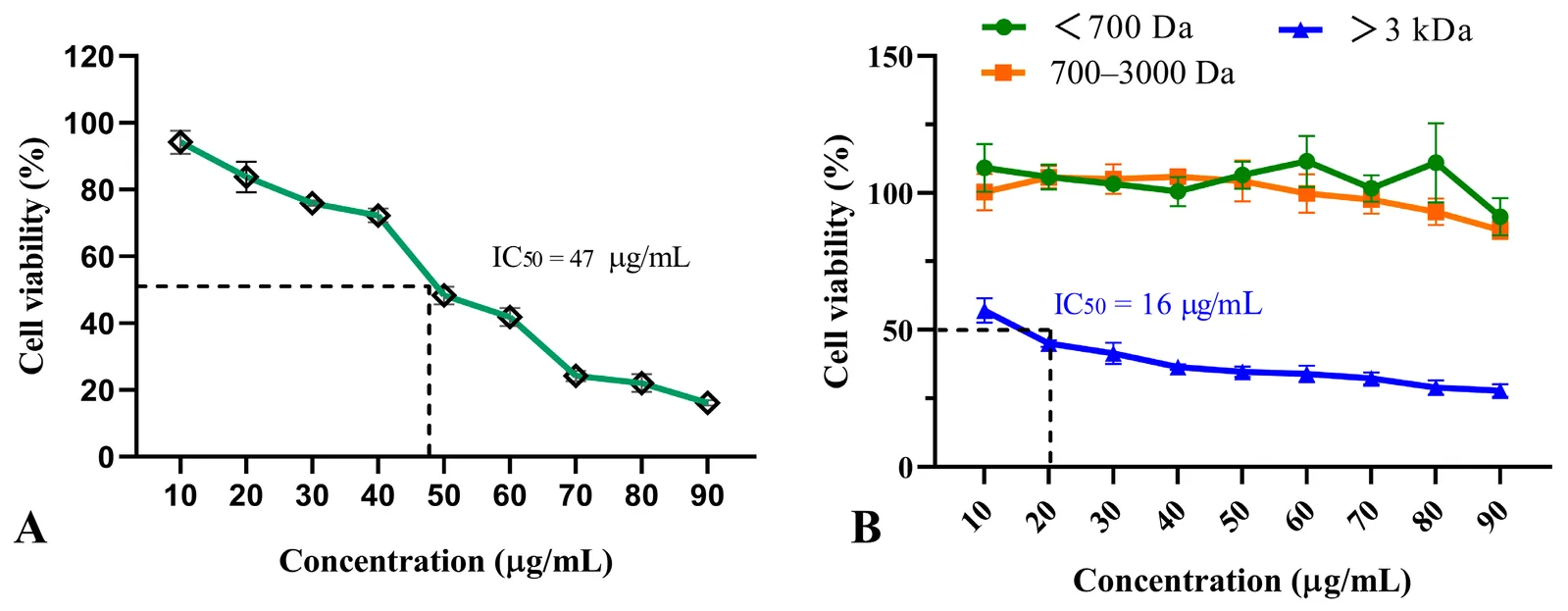
THLE-2 cytotoxicity evaluation of VBsV and its molecular-weight fractions. (**A**), Effect of VBsV at different concentrations on the viability of THLE-2 cells; (**B**), effect of each molecular-weight-fractionated component on the viability of THLE-2 cells. Cell-based experiments were conducted in triplicate, with six technical replicates per experiment. The black dashed lines indicate the 50% cell-viability level and the corresponding IC_50_ estimate.

THLE-2 cells were used to evaluate the in vitro cytotoxicity of crude VBsV and its molecular-weight fractions. As the concentration of crude VBsV increased from 10 to 90 μg/mL, THLE-2 cell viability decreased in a concentration-dependent manner, with an IC_50_ of 47 μg/mL (Figure 5A), indicating a significant inhibitory effect on normal liver cells.

Further comparison of the molecular-weight fractions showed that neither the <700 Da fraction nor the 700–3000 Da fraction markedly inhibited THLE-2 cell viability at 10–80 μg/mL, with cell viability generally remaining above approximately 90% (Figure 5B). At 90 μg/mL, both fractions caused a certain degree of reduction in cell viability. In contrast, the >3 kDa fraction displayed pronounced cytotoxicity at all tested concentrations, and cell viability declined further as the concentration increased, with an IC_50_ of 16 μg/mL.

## 3. Discussion

This study combined proteomics, metabolomics, molecular-weight-directed fractionation, and in vitro activity and cytotoxicity evaluation techniques to systematically analyze the chemical composition and protein composition of the VBsV. It clearly revealed significant differences in biological activity and cytotoxicity effects among different molecular-weight components, providing a new experimental basis and theoretical perspectives for the study of the component-activity relationship of natural toxins from vespa venom. Within the tested concentration ranges, the 700–3000 Da fraction retained concentration-dependent NO inhibition and tumor-cell-inhibitory activities while exhibiting relatively low cytotoxicity toward THLE-2 cells. The >3 kDa fraction also displayed pronounced activity at low concentrations, but this was accompanied by substantial cytotoxicity. In contrast, the <700 Da fraction showed neither target activity nor evident cytotoxicity in vitro.

Proteomic analysis identified the major protein constituents of VBsV, including two phospholipase A1 (PLA1) isoforms corresponding to proteins 3 and 4, hyaluronidase A corresponding to protein 2, an antigen 5 family protein, and a venom-derived dipeptidyl peptidase corresponding to protein 1. Previous studies have established PLA1, hyaluronidase A, and antigen 5 as major allergens in vespa venom and have demonstrated varying degrees of IgE-mediated cross-reactivity among these proteins [20,21,22].

Although antigen 5 itself does not exhibit direct cytotoxicity, it can induce a strong type I hypersensitivity response in vivo [9,23]. Hyaluronidase A is a classical “spreading factor” in insect venom. By degrading hyaluronic acid in the extracellular matrix, it markedly increases tissue and vascular permeability and thereby facilitates the rapid diffusion of other venom components. Hyaluronan oligosaccharides generated by this hydrolysis have also been shown to possess pronounced pro-inflammatory and immunostimulatory activities [2,24,25]. Homologs of venom dipeptidyl peptidase have been reported to be major allergens in vespa venom; they can efficiently cross-link receptors and activate basophils from sensitized patients, thereby triggering severe systemic anaphylactic reactions [26,27]. PLA1, a major allergenic protein in VBsV, has been reported to possess hemolytic activity and to directly disrupt cell-membrane structures, resulting in severe hemolysis and cardiac dysfunction [1]. It can also specifically hydrolyze the sn-1 ester bond of phosphatidylserine (PS) in cell membranes to generate lysophosphatidylserine (lysoPS) [28,29] which can subsequently induce allergic reactions and acute inflammation [30].

These findings indicate that the >3 kDa fraction of VBsV contains several classical components capable of inducing allergic and inflammatory responses in vivo. The results may therefore provide a reference for the future screening of immunodiagnostic markers and for allergen-specific immunotherapy targeting vespa venom. In addition, the four characteristic bands that consistently appeared between 25 and 116 kDa in the SDS-PAGE profiles of VBsV displayed highly reproducible migration positions, relative abundances, and band numbers. This pattern may provide a preliminary reference for the development of a characteristic protein fingerprint for the identification and quality control of vespa-venom samples.

In addition to proteins, VBsV exhibited a complex profile of low-molecular-weight metabolites, further illustrating the chemical complexity of vespa venom. Targeted neurotransmitter analysis showed relatively high normalized abundances of 5-HT, L-glutamate, histamine, and dopamine. Histamine induces vasodilation, increases vascular permeability, and promotes local inflammatory responses [31]. 5-HT has been implicated in the pain and neural stimulation induced by hymenopteran venoms [32], whereas glutamate is an important excitatory neurotransmitter that may participate particularly in venom-mediated effects on the insect neuromuscular system [33,34]. These neuroactive or vasoactive compounds may contribute to pain, neural stimulation, altered vascular permeability, and local inflammation after a hornet sting, thereby enhancing the defensive effects of the venom. At the same time, the detection of GABA, adenosine, and inosine suggests that VBsV may also contain constituents associated with inhibitory neural signaling and anti-inflammatory activity. However, the <700 Da fraction did not significantly inhibit LPS-induced NO release from RAW264.7 cells in the present study, indicating that these low-molecular-weight metabolites were not the principal contributors to the NO-suppressive effect observed in this model. Their roles may instead be more closely related to pain, neural stimulation, and local vascular responses. Notably, the increased CCK-8 signals observed at some concentrations cannot be assigned to metabolic substrate provision or hormesis from the current data; their biological basis remains unknown. Moreover, no individual detected metabolite was experimentally linked to any cell-based endpoint.

In the present study, VBsV exhibited pronounced NO inhibition and tumor-cell-inhibitory activities in vitro; however, crude venom also showed marked cytotoxicity toward THLE-2 cells. Further fractionation revealed that the 700–3000 Da and >3 kDa fractions displayed the greatest activity under the tested conditions, whereas cytotoxicity toward normal liver cells was mainly distributed in the >3 kDa fraction. This finding suggests a distinct activity–risk partition among fractions of different molecular weights. We speculate that the cytotoxic effect observed in both tumor cells and normal liver cells by the >3 kDa fraction may be related to the membrane-disrupting activity of phospholipase A_1_ (PLA_1_) contained within it. Notably, this fraction still showed significant NO inhibition at low concentrations that did not markedly affect RAW264.7 cell viability, a finding that is not fully consistent with the traditionally recognized pro-inflammatory (NO levels increased), hemolytic, or allergenic activities of its known proteins. Previous research has suggested that mammalian phosphatidylserine-specific PLA1 (PS-PLA1) can exert context-dependent anti-inflammatory effects by downregulating the phosphorylation of p38, ERK, and JNK and thereby inhibiting MAPK signaling, raising the possibility that PLA1 in VBsV may be a contributing protein [35]. However, because the >3 kDa fraction remains a mixed protein system, its in vitro NO inhibition effect cannot currently be unequivocally attributed to VBsV PLA1 or any other specific protein. It also remains possible that PLA1 or other proteins in VBsV possess distinctive biological activities. Their actual contributions require verification using purified proteins, enzyme-activity inhibition, or recombinant-protein experiments. These results provide important clues and directions for the future discovery of novel functional proteins.

By comparison, the 700–3000 Da fraction largely reproduced the NO inhibition effect and tumor-cell-inhibitory activities of crude venom while exerting a relatively limited effect on THLE-2 cells, thus showing a more favorable preliminary in vitro activity–cytotoxicity profile. Although the omics analysis in this study identified only one peptide, mastoparan-like peptide 12a, this does not imply that the fraction contained only a single bioactive peptide. The result may have been influenced by peptide abundance, ionization efficiency, database coverage, and identification thresholds. Previous studies have isolated mastoparan B [12,16], HP-series peptides including HP-1, HP-2, and HP-3 [19], and recently identified nociceptive peptide components, including components 1-1, 1-2, and 9 [13], from VBsV, all of which have molecular weights ranging between 700 and 3000 Da. However, current research has primarily focused on their hemolytic, edema-inducing, pain-producing, and other acute toxic effects, while knowledge regarding their anti-inflammatory and tumor-cell-inhibitory potential remains limited. Thus, the significance of this study lies not in being the first to demonstrate the presence of active peptides in VBsV or to attribute biological activity to a specific peptide, but rather in using an activity-guided strategy to identify active compounds in vespa venom within the 700–3000 Da range and to preliminarily reveal their more favorable in vitro activity–cytotoxicity profile compared with that of the >3 kDa fraction. These findings provide important research clues and developmental directions for the subsequent pharmaceutical development of bioactive peptides from VBsV.

This study has several limitations. First, each molecular-weight fraction is a complex mixture obtained through operational separation; even with proteomic and metabolomic analyses conducted to identify its components, it remains unclear which specific component contributes to the observed NO inhibition and cytotoxic effects. Second, targeted analysis of neurotransmitters and energy metabolites was conducted based on a single representative batch, and the results are limited to qualitative identification of compounds and relative abundance comparisons and cannot be used for absolute quantification of components. Third, the NO and CCK-8 assays assessed only LPS-induced NO production and cellular metabolic activity as a proxy for cell viability, respectively; therefore, these data do not establish broad anti-inflammatory activity, antitumor efficacy, or tumor-selective cytotoxicity. Fourth, normal-cell evaluation was limited to THLE-2 cells, and hemolysis, mast-cell degranulation, pain, allergenicity, and in vivo toxicity have not yet been examined. Moreover, the activity evaluations in this study were based on in vitro cell models, and the results reflect pharmacological effects under specific conditions, which cannot yet be extrapolated to in vivo physiological environments or clinical efficacy. Accordingly, the 700–3000 Da fraction can currently be regarded only as a priority fraction with a relatively favorable in vitro activity–cytotoxicity profile. Its pharmacological value and overall safety require further validation.

## 4. Conclusions

Combined compositional profiling and operational molecular-weight fractionation revealed distinct in vitro profiles among VBsV fractions. At equal nominal mass concentrations, the 700–3000 Da and >3 kDa fractions most strongly inhibited LPS-induced NO production and reduced tumor-cell viability. The 700–3000 Da fraction showed concentration-dependent effects and lower THLE-2 cytotoxicity than the >3 kDa fraction at 10–80 μg/mL. Because the fractions remain mixtures and total activity recovery was not determined, the responsible molecules and therapeutic relevance remain unresolved. Direct characterization and purification of the 700–3000 Da fraction are the next priorities.

## 5. Materials and Methods

### 5.1. Sample Collection and Preparation

VBsV samples were collected in Wenshan City, Yunnan Province, China, between 2022 and 2024. Species identification was performed by Professor Yunjiao Guo of the Institute of Edible Insects, Dehong Normal University, based on morphological characteristics and taxonomic criteria, and the specimens were confirmed as *V. basalis* Smith. VBsV was collected by pulsed electrical stimulation. The crude venom was resuspended in precooled ultrapure water and centrifuged at 5000× *g* for 5 min at 4 °C. The supernatant was collected, lyophilized to obtain crude venom powder, sealed, and stored at −20 °C until use.

This study did not involve humans or vertebrate animals. *Vespa basalis* is an invertebrate insect, and formal animal ethics approval was not required under the institutional regulations applicable to this study.

### 5.2. Proteomic Analysis

#### 5.2.1. Molecular-Mass Determination of Venom Proteins

VBsV was separated by 15% sodium dodecyl sulfate–polyacrylamide gel electrophoresis (SDS-PAGE) using SDS-PAGE reagents (Beyotime Biotechnology, Shanghai, China) and visualized using Coomassie Brilliant Blue staining solution (Beyotime Biotechnology, Shanghai, China), as previously described [10]. Lyophilized crude venom was dissolved in ultrapure water before electrophoresis. Apparent molecular masses were estimated by comparison with the protein molecular-mass marker (Beyotime Biotechnology, Shanghai, China).

Intact-protein molecular-mass measurements were performed by matrix-assisted laser desorption/ionization time-of-flight mass spectrometry (MALDI-TOF-MS; ultrafleX-treme, Bruker Daltonics, Bremen, Germany) using the previously reported approach [10]. Sinapinic acid (Merck, Darmstadt, Germany) was used as the matrix. Spectra were acquired in positive-ion linear mode over *m*/*z* 10,000–100,000; ion-source voltages were 20 kV (IS1) and 18.9 kV (IS2), and the lens voltage was 6.85 kV.

#### 5.2.2. Global Identification of VBsV

VBsV was reconstituted to 1 mg/mL, and low-molecular-weight compounds were removed using a G-10 Mini desalting column (Beyotime Biotechnology, Shanghai, China). After reduction, alkylation, and tryptic digestion, the samples were desalted using a Sep-Pak C18 cartridge (Waters, Milford, MA, USA). The peptide mixture was injected into a liquid chromatography system (Thermo Fisher Scientific, Waltham, MA, USA) equipped with a nano-trap column (Acclaim PepMap C18, 100 µm × 20 mm) and an analytical column (Acclaim PepMap C18, 75 µm × 250 mm). Mobile phase A consisted of 0.1% formic acid (Merck, Darmstadt, Germany) in water, and mobile phase B consisted of 0.1% formic acid in acetonitrile (Merck, Darmstadt, Germany). The gradient was programmed as follows: 0–45 min, 0–45% B; 45–55 min, 45–80% B; and 55–60 min, 80% B. The flow rate was 350 nL/min.

Mass spectrometric detection was performed using a Q Exactive LC–MS/MS system (Thermo Fisher Scientific, Waltham, MA, USA) equipped with an electrospray ionization source and operated in a Top-20 data-dependent acquisition mode. The resolutions of full-scan MS and MS/MS were 70,000 and 17,500, respectively. The scan ranges for both full MS and dd-MS2 were 350–1800 *m*/*z*.

Raw data were processed using Proteome Discoverer 2.1 with the Sequest HT search engine and searched against *Vespa* entries in the UniProt database. The identification parameters were set as follows: peptide mass tolerance, 10 ppm; MS/MS tolerance, 0.02 Da; fixed modification, carbamidomethylation; and variable modification, oxidation. A false discovery rate (FDR) of ≤0.01 was used as the filtering criterion for all peptide and protein identifications.

#### 5.2.3. Identification by In-Gel Digestion

Target protein bands unresolved in Section 5.2.2—including proteins 1, 2, and 3—were excised from the Coomassie-stained SDS-PAGE gel and subjected to in-gel tryptic digestion based on the established protocol of Shevchenko et al. [36], using sequencing-grade trypsin (Promega, Madison, WI, USA). After peptide extraction and de-salting, samples were analyzed using a Q Exactive Plus LC–MS/MS system (Thermo Fisher Scientific, Waltham, MA, USA). Chromatographic separation used a custom-packed C18-AQ capillary column (1.9 μm, 75 μm internal diameter; Dr. Maisch GmbH, Ammerbuch, Germany). Mobile phase A was 0.1% formic acid in water and mobile phase B was 0.1% formic acid in acetonitrile/water (80:20, *v*/*v*). The gradient was 0–2 min, 3–5% B; 2–47 min, 5–40% B; 47–52 min, 40–100% B; and 52–60 min, 100% B at 300 nL/min. The full MS range was 355–2000 *m*/*z* and the fixed first mass for dd-MS2 was 120 *m*/*z*. Other mass spectrometric and database-search settings matched Section 5.2.2.

### 5.3. Metabolomic Profiling

#### 5.3.1. Untargeted Metabolomic Analysis

Venom samples were reconstituted in ultrapure water to a final concentration of 1 mg/mL and submitted to Hangzhou Lianchuan Biotechnology Co., Ltd. (Hangzhou, China) for untargeted metabolomic analysis. Data were acquired on an LC-MS/MS platform in both positive- and negative-ion modes. Raw mass spectrometric data were processed using XCMS for peak extraction, retention-time correction, and signal alignment. Metabolite identification and annotation of characteristic ion features were subsequently performed against the Human Metabolome Database (HMDB) and the Kyoto Encyclopedia of Genes and Genomes (KEGG) database.

#### 5.3.2. Targeted Metabolomic Analysis

Venom samples were reconstituted in ultrapure water to a final concentration of 1 mg/mL. Proteins were precipitated with methanol, and the resulting supernatants were submitted to Hangzhou Lianchuan Biotechnology Co., Ltd. (Hangzhou, China) for targeted metabolomic analysis of 154 energy metabolites and 21 neurotransmitters.

The 154 energy metabolites were semiquantitatively profiled in positive- and negative-ion modes using an Agilent 1290 ultrahigh-performance liquid chromatography (UPLC) system (Agilent Technologies Inc., Santa Clara, CA, USA) coupled to an Agilent 6545 Q-TOF high-resolution mass spectrometer (Agilent Technologies Inc., Santa Clara, CA, USA) equipped with an AJS electrospray ionization source. Chromatographic separation was performed on a hydrophilic-interaction column (Waters BEH Amide, 2.1 × 100 mm, 1.7 μm; Waters, Milford, MA, USA). Mobile phase A consisted of water containing 15 mM ammonium acetate (CNW, ANPEL Laboratory Technologies (Shanghai) Inc., Shanghai, China) and 0.3% aqueous ammonia (CNW, ANPEL Laboratory Technologies (Shanghai) Inc., Shanghai, China), whereas mobile phase B consisted of 90% aqueous acetonitrile (CNW, ANPEL Laboratory Technologies (Shanghai) Inc., Shanghai, China) containing 15 mM ammonium acetate and 0.3% aqueous ammonia. The column temperature was 40 °C, and the injection volume was 2 μL. The mass spectrometric parameters were as follows: drying-gas temperature, 300 °C; sheath-gas temperature, 325 °C; drying-gas flow rate, 8 L/min; sheath-gas flow rate, 11 L/min; nebulizer pressure, 40 psi; capillary voltage, 3500 V; fragmentor voltage, 110 V; and nozzle voltage, 500 V in positive-ion mode and 1000 V in negative-ion mode.

For the analysis of 21 neurotransmitters, chromatographic separation was carried out using a Vanquish reversed-phase liquid chromatography system (Thermo Fisher Scientific, Waltham, MA, USA) equipped with an Agilent Eclipse Plus C18 column (3.0 × 150 mm, 1.8 μm; Agilent Technologies Inc., Santa Clara, CA, USA). The column temperature was maintained at 30 °C, and the injection volume was 1 μL. Mobile phase A was 0.6% formic acid in water, and mobile phase B was acetonitrile. Mass spectrometric detection was performed using a TSQ Altis triple-quadrupole mass spectrometer (Thermo Fisher Scientific, Waltham, MA, USA) equipped with an electrospray ionization source. Quantitative analysis was performed using multiple reaction monitoring (MRM). The ion-source temperature was 350 °C, the spray voltage was 3500 V, the sheath gas was set to 30 Arb, the auxiliary gas to 25 Arb, and the sweep gas to 0 Arb. MRM parameters were optimized for each compound. All target analytes were qualitatively confirmed by comparing their retention times and MRM ion transitions with those of authentic standards (purity >99%; Tianjin ALTA Technology Co., Ltd., Tianjin, China). Quantification was performed using an external-standard method, with calibration curves constructed by plotting the concentration of each compound against the peak area of the corresponding MRM channel. VBsV analysis was performed on a single representative batch solely for compound identification and relative abundance comparison; absolute quantification was not attempted.

### 5.4. Preliminary Fractionation and Purification of VBsV

#### Ultrafiltration and Desalting by Gel-Filtration Chromatography

VBsV was reconstituted in ultrapure water to a concentration of ~20 mg/mL and subjected to centrifugal ultrafiltration using a 3-kDa molecular-weight cutoff (MWCO) device (Merck, Darmstadt, Germany), yielding fractions >3 kDa and <3 kDa. The <3 kDa fraction was subsequently subjected to gel-filtration chromatography on a Sephadex G-10 column (OriLeaf, Shanghai, China; 1.6 cm × 30 cm) for desalting and further fractionation, yielding an operationally defined 700–3000 Da fraction and a <700 Da fraction. Precooled ultrapure water was used as the mobile phase at a flow rate of 1 mL/min, and fractions were collected every 3 min. Elution was monitored at 280 and 595 nm. The target peaks were collected, lyophilized, and stored at −80 °C. The >3 kDa fraction was characterized by SDS-PAGE as described in Section 5.2.1. The 700–3000 Da fraction was separately analyzed by MALDI-TOF-MS using α-cyano-4-hydroxycinnamic acid (HCCA) as the matrix in positive-ion mode. The mass spectrum was acquired over an *m*/*z* range of approximately 700–3500 to examine the intact-mass distribution of detectable constituents within this fraction.

### 5.5. Cell Culture

RAW264.7 mouse macrophages, THLE-2 human liver epithelial cells, and the indicated human tumor-cell lines (HepG2, MCF-7, RKO, A549, HeLa, THP-1, and K562) were obtained from the American Type Culture Collection (ATCC). THLE-2 cells were maintained in a dedicated THLE-2 medium (BDBIO, Zhejiang, China). THP-1 and K562 cells were cultured in RPMI-1640 medium supplemented with 10% fetal bovine serum (FBS), whereas the remaining cell lines were maintained in high-glucose DMEM containing 10% FBS. Cells were cultured in a humidified incubator at 37 °C with 5% CO_2_, and all treatment incubations were conducted under the same environmental conditions unless otherwise stated. Cell-culture waste generated during the experiments was segregated from routine laboratory waste and disposed of through the institutional biological-waste management system. General laboratory waste-management guidance was referenced to the WHO Laboratory Biosafety Manual [37].

### 5.6. In Vitro NO-Suppression and Cell-Viability Assays

#### 5.6.1. LPS-Induced NO Production and Concurrent RAW264.7 Viability

RAW264.7 cells (2 × 10^4^ cells/well) were stimulated with 1 μg/mL LPS. Complete medium served as the unstimulated control, and 10 μg/mL dexamethasone (Biosharp, Beijing, China) served as the positive control. VBsV or its operationally defined >3 kDa, 700–3000 Da, and <700 Da fractions were added for a 24 h exposure. The supernatants were subsequently collected, and NO concentrations were quantified using an NO detection kit (Biosharp, Beijing, China). In parallel, RAW264.7 metabolic viability was measured by CCK-8 under the same treatment concentrations and 24 h exposure conditions to determine whether the reduction in NO production could be explained by overt cytotoxicity. The NO-inhibition rate was calculated according to the following equation: NO inhibition (%) = [A(model) − A(sample)]/[A(model) − A(control)] × 100%. Accordingly, the LPS model and unstimulated control were defined as 0% and 100% inhibition, respectively. The experimental concentration–inhibition data were analyzed by probit regression analysis using IBM SPSS Statistics 27.0.1. The concentration predicted by the regression model to produce 50% inhibition of LPS-induced NO production was reported as the NO-suppression IC_50_.

#### 5.6.2. Tumor-Derived and THLE-2 Cell-Viability Assays

The effects of VBsV on cell viability were evaluated by CCK-8 assays in HepG2, MCF-7, RKO, A549, HeLa, THP-1, and K562 tumor-derived cells. HeLa and THP-1 cells were selected for subsequent comparisons of the molecular-weight fractions. THLE-2 cells were used as a single non-malignant cell model for preliminary cytotoxicity comparison. Cells were seeded at 1 × 10^4^ cells/well for 24 h, exposed to Vs. or its fractions for an additional 24 h, and then incubated with CCK-8 reagent in the dark for 1–2 h. Absorbance was measured at 450 nm. Untreated cells were defined as 100% viability, and cell viability was calculated as A(sample)/A(untreated control) × 100%. The experimental concentration–viability data were analyzed by probit regression analysis using IBM SPSS Statistics 27.0.1. The concentration predicted by the regression model to reduce the normalized CCK-8 viability signal to 50% of the untreated control was reported as the cell-viability IC_50_. Comparisons between tumor-derived and THLE-2 cells were descriptive and do not establish tumor selectivity or overall safety.

### 5.7. Statistical Analysis

Unless otherwise stated, cell-based experiments were performed in three independent experiments, each with 6 technical replicate wells, and data are presented as the mean ± standard deviation (mean ± SD). Statistical analyses were conducted using GraphPad Prism 9.0 and IBM SPSS 27.0.1. Comparisons among multiple groups were performed using one-way analysis of variance (ANOVA), followed by Tukey’s multiple-comparison test [38,39]. Statistical significance was defined as * *p* < 0.05, ** *p* < 0.01, *** *p* < 0.001, and **** *p* < 0.0001.

## 6. Patent

A Chinese patent application arising from this work has been filed: A method for preparing low-pain and low-allergenic vespa venom active peptides and its applications. Application number: 2026110409154.

## Data Availability

The mass spectrometry proteomics data generated in this study have been deposited with the ProteomeXchange Consortium via the iProX partner repository under the dataset identifier PXD082039 and iProX accession IPX0018755000.

## Supplementary Materials

The following supporting information can be downloaded at https://www.mdpi.com/article/10.3390/toxins18090394/s1, Figure S1. LC–MS/MS identification of four major VBsV proteins. Matched peptide sequences are highlighted in the reference sequences, and one representative MS/MS spectrum is shown for each protein: (A) venom dipeptidyl peptidase IV (protein 1), (B) hyaluronidase A (protein 2), (C) a phospholipase A1 isoform (protein 3), and (D) phospholipase A1 (protein 4). Figure S2. Allergen 5 protein (A) and mastoparan-like peptide 12a (B) of VBsV were identified by LC-MS/MS. The sequence coverage of the matched peptides is highlighted, and the representative MS/MS spectra are displayed below. Figure S3. Total ion chromatograms (TICs) of untargeted metabolomics for three batches of VBsV samples: A, positive-ion mode; B, negative-ion mode. Figure S4. MALDI-TOF-MS profile of the 700–3000 Da fraction of VBsV. Prominent ions were detected at *m*/*z* 1337.8457, 1470.9259, 1652.9930, 1933.0086, and 2927.4561. The intact-mass profile supports the presence of detectable ions within the intended range but does not establish their chemical identities or peptide nature. Table S1. Targeted mass spectrometry analysis of the relative abundance of 21 neurotransmitters in VBsV. Table S2. Targeted mass spectrometry analysis of the relative abundance of 154 energy metabolites in VBsV. Supplementary Data S1. 720 putatively annotated LC–MS/MS features.
