# Supplementary material for: Composition and In Vitro Activity–Cytotoxicity Profiles of Vespa basalis Venom: Combined Proteomic, Metabolomic, and Molecular-Weight-Fractionated Analyses
Source: Toxins (Basel). 2026 Sep 12;18(9):394. doi: 10.3390/toxins18090394 (PMC13611018; doi:10.3390/toxins18090394)
Supplement: Supplementary file 1 [file toxins-18-00394-s001.zip › Supplementary Material.pdf]

Protein 1: Venom dipeptidyl peptidase IV

MVPLRSFVLL NGLFFVLLAA RTVVTR**VIDK DNLDGILETQ DCQNLSK**EPF NLEETYTADE

LAYTFNGTWT SDTTIVYTDT MTGDILQFDV IKQRLTVIVD SSVMDYIVT HYALSPKGR**F**

LLIGYDFQKG FRHSKFMR**YV IYDIELGGYD KIANGMHIAL AKWAPLTDLL IYILNDIYY**

MRFSNNGFND VQRVTYDGIV GIVYNGVPDW VYEEEVEHGS SAIWFSPDGS HLAYASFDDR

NVOEILYLHY GEPGNLVDQY PTEVKIKYPK AGTSNPVVSL TLVDLHDPTL NKIDLK**APIE**

VVGTDNVLSN VQWKDFDHVI AMWSNRVQNK **TEIVVWNK**YG EIVK**TLDVVE HEGWVEIKNL**

FFYKDFVMR **KLQPSGTK**AG R**FHHVTR**YDY TLRSSPIQMD LTPGITEVQD IRAIDHSHGR

IYYLATGPGE PSQRNLYSVP VDGSEKPTCI SCNVLTPEGN ACTYADAIFS PFGQHVYLIC

QGPDMIVGI FDNTKHKVYS WENNLSLRSK LARRELPLVK DLVVRANGYE SKVR**LFLPHN**

FDESKSYPML VNVYAGPNTA KIIDVASVGY HAYMTNRSV IYAYIDGRGS SNKGSK**MLFE**

IYRKLGTVEV EDQISVTRKL QEMFPWIDSK RTGVWGSY GGFCSAMILAK DLTSVFK**CGI**

AVAPVSSWIY YDSIYTERYM GLPTPEDNLG GYNGTDVSRR VEDIRGK**KFM LIHGSGDDNV**

HYQOSLALAK ALEK**ADIMFE QITYTDEAHA LFGVLPLHYH TMDR**FWSDCF SLSHAH

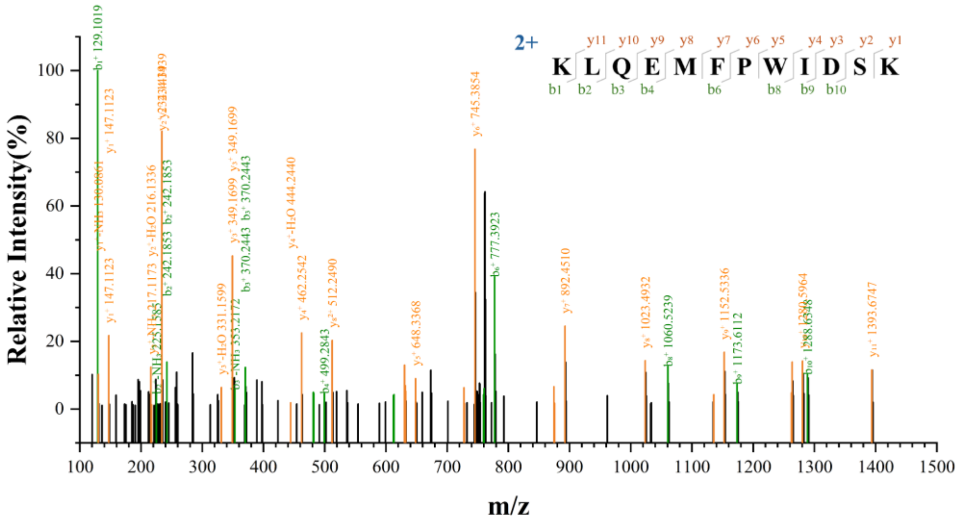

A

Protein 2: Hyaluronidase A

NLNRTNWPKK IFNIYWNVPT YFCHQHDVYF NELTKFDIKY NPKGNYRGDT ISLFYDPGNF

PAMVPLKNGT YDIR**NEGVPQ K**GNITVHLQQ FTK**ELDEIYP KKISGGIGVI NFNKWRPIFR**

RNVNLKINK EVSIDLVRKE HPKWDKSMIE TEASNRFEKS ARIFMEKTLK LAKDIRNKNK

WGYHGYPYCP TASTGNPSFD CDALAMNEND KLSWLFKYQD VLLPSVYVK**H VLKPEEK**IGL

VRGSVKEAVR **ISKKFEHLPK VLSYWWYAYE DKMDTFLTET DVK**NTFREIL INGGDGIIIW

GTMHDLNKEK CEK**LKQYLST ILGPIAFK**VM EAVKRRTPLN F

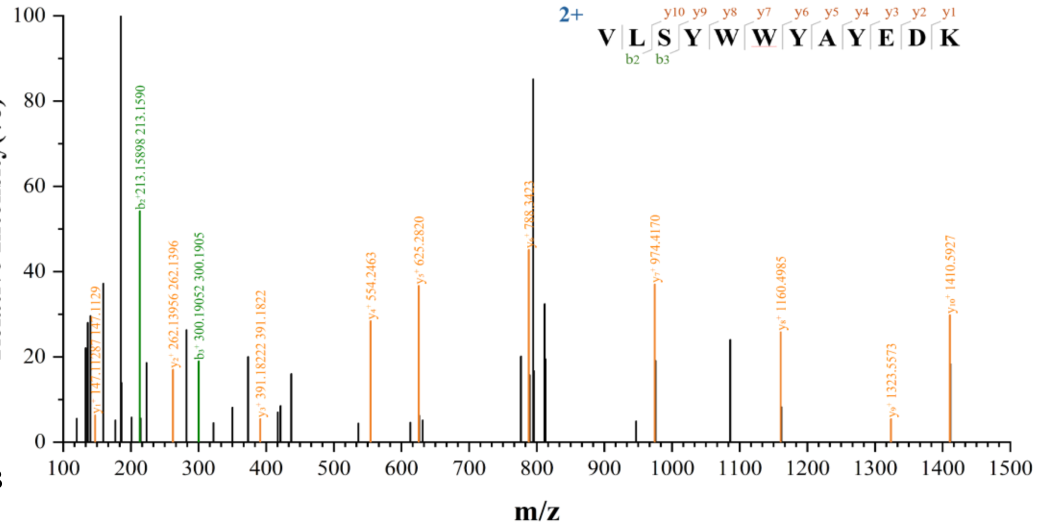

B

Protein 3: Phospholipase A1

ENPCPYSDDT VKMIILTREN K**KHDFYTLDT IKK**HNEFKKS TIK**HQVVFIT HGFTSSADTE**
NFLAMAKALS DKGNYLVILI DWRVAACTEE MSGIQLAYYS YAASNTR**LVG NYIATVTKML**
VQKYNVPMAN IRLIGHSLGA HTSGFAGKKV QELGLGKYSE IIGLDPAGPS FKSNDCSERI
CKTDAHYVQI IHTSNHLGTL VTLGTVDEMN NGYNQPGCGL PLIGETCSHT RAVKYFTECI
K**HECCLIGVP QSK**KPQPVSK CTR**NECVCVG LNAKTYPKTG SFYVPVESK**A PYCNNKGKII

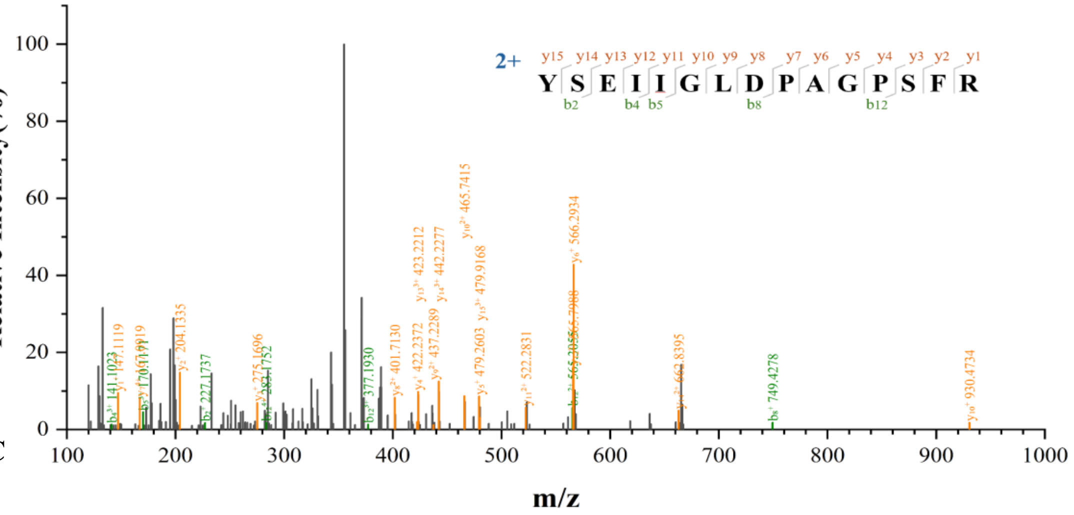

C

Protein 4: Phospholipase A1

FNPCPYSDDT VKMIILTREN KKHDFYTLDT IKKHNEFKKS TIKHQVVFIT HGFTSSADTE
NFLAMAKALS DKGNYLVILI DWRVAACTEE MSGIQLAYYS YAASNTR**LVG NYIATVTKML**
VQKYNVPMAN IRLIGHSLGA HTSGFAGKKV QELGLGKYSE IIGLDPAGPS FKSNDCSERI
CKTDAHYVQI IHTSNHLGTL VTLGTVDFMN NGYNQPGCGL PLIGETCSHT R**AVKYFTECI**
KHECCLIGVP QSKKPQPVSK CTRNECVCVG LNAKTYPKTG SFYVPVESKA PYCNNKGKII

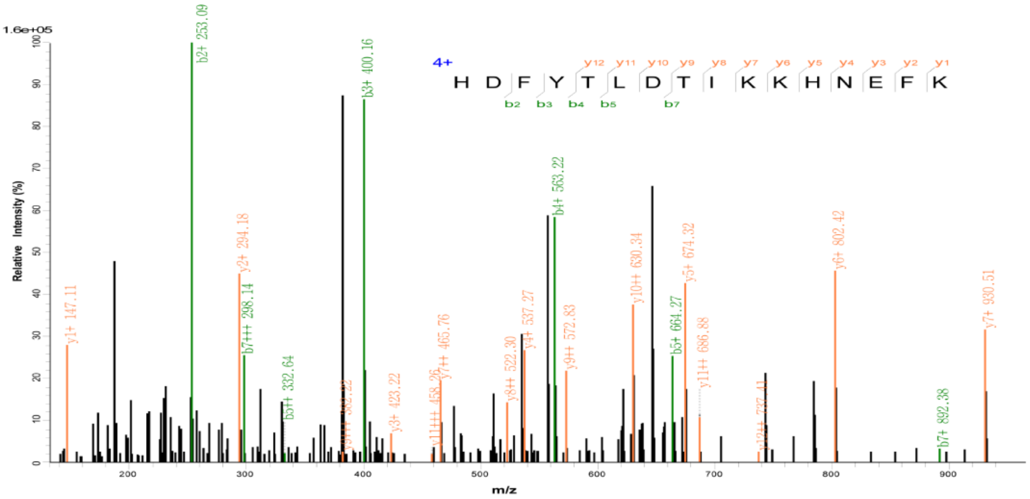

D

Figure S1. LC–MS/MS identification of four major VBsV proteins. Matched peptide sequences are highlighted in the reference sequences, and one representative MS/MS spectrum is shown for each protein: (A) venom dipeptidyl peptidase IV (protein 1), (B) hyaluronidase A (protein 2), (C) a phospholipase A1 isoform (protein 3), and (D) phospholipase A1 (protein 4).

Venom allergen 5

NNYCKIKCRS GIHTLCKYGT STKPNCGRSV VKASGLTKAE KLEILKQHNE FRQKVARGLE TRGNPGPQPP AKSMNTLVWN DELAQIAQVW ASQCKYGHDN CRNTAK**YLVG QNIAEQSTTA ASFEPVSNMV K**MWSDEVKDY QYGSSK**NKLN DVGHYTQMVW AK**TKEIGCGN IK**YIENGWHH HYLVCNYGPA GNIGNEPIYE KK**

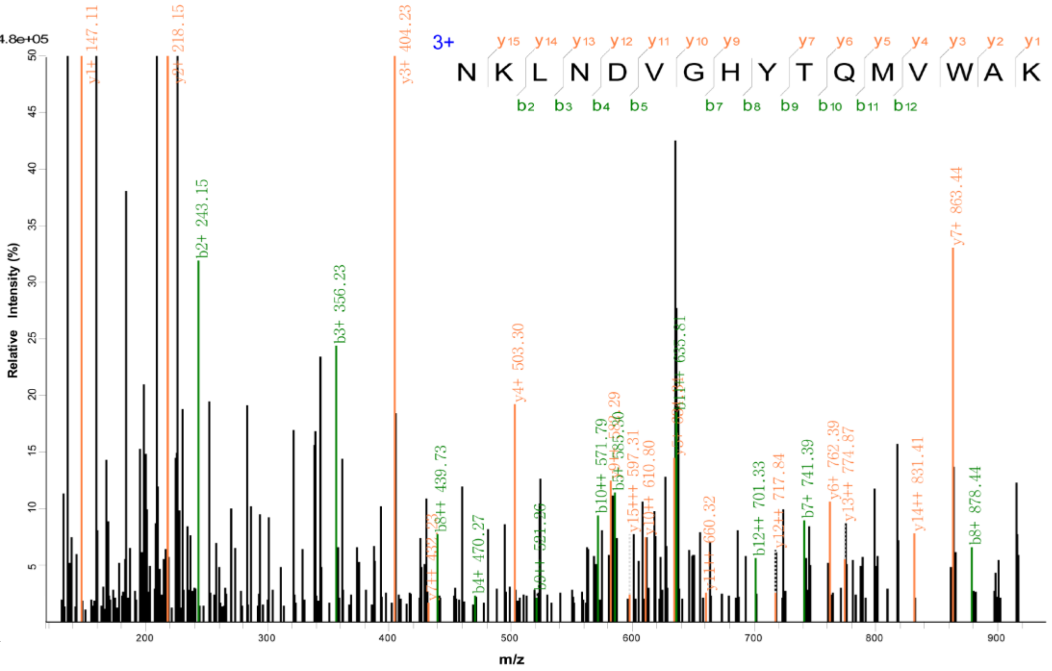

A

Mastoparan-like peptide 12a

INWKGIAAMA KKLL

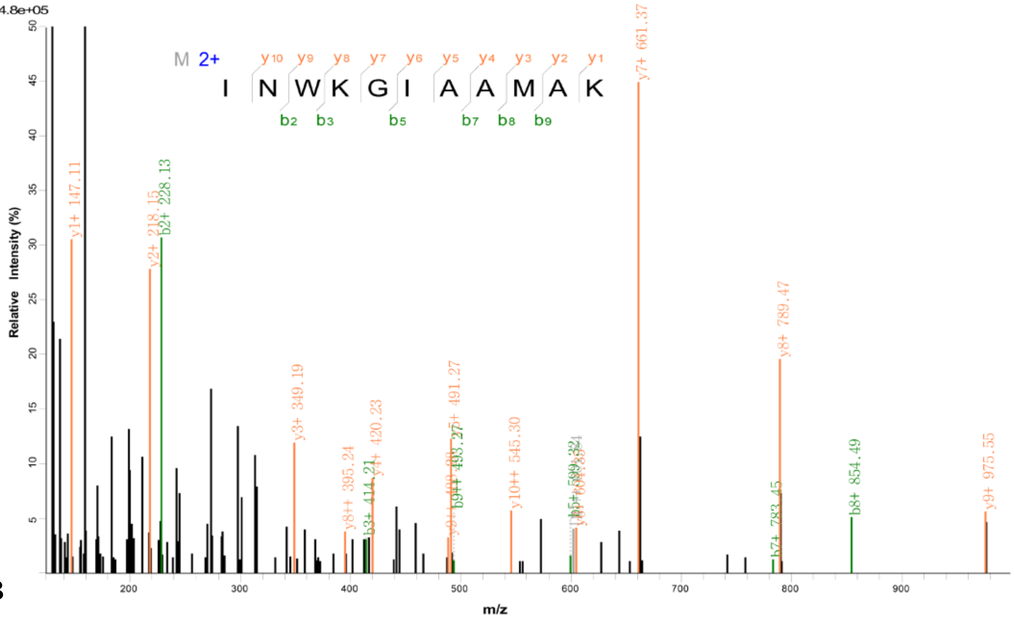

B

Figure S2. Allergen 5 protein (A) and mastoparan-like peptide 12a (B) of VBsV were identified by LC-MS/MS. The sequence coverage of the matched peptides is highlighted, and the representative MS/MS spectra are displayed below.

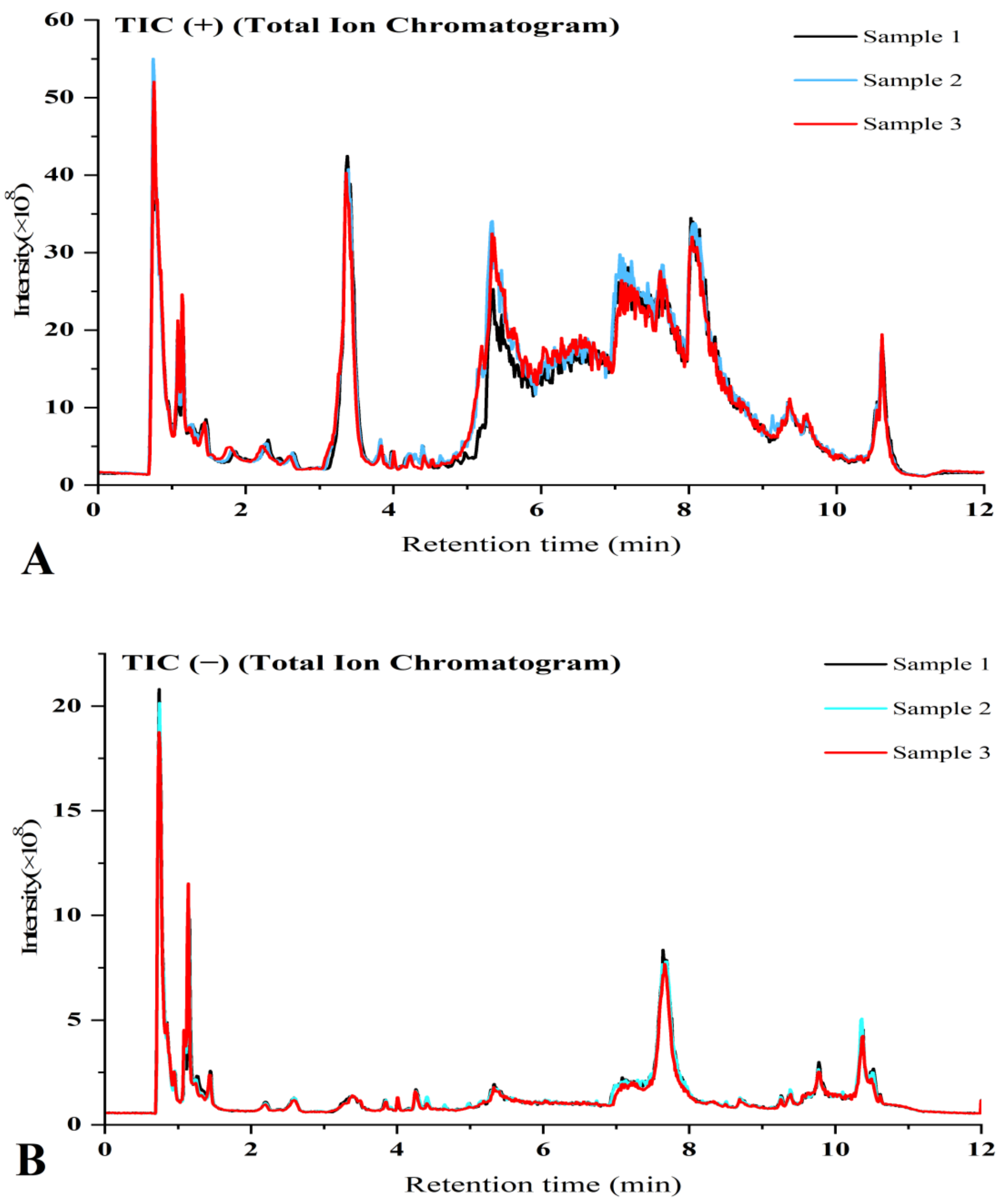

Figure S3. Total ion chromatograms (TIC) of untargeted metabolomics for three batches of VBsV samples: A, positive ion mode; B, negative ion mode

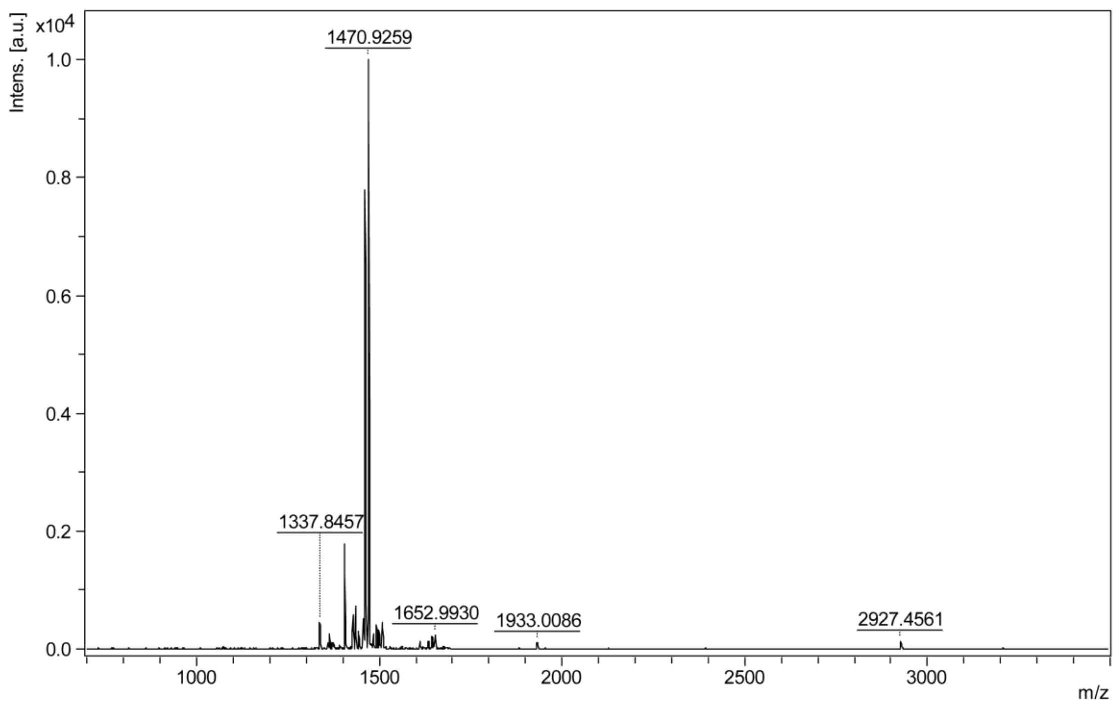

Figure S4. MALDI-TOF-MS profile of the 700–3000 Da fraction of VBsV. Prominent ions were detected at m/z 1337.8457, 1470.9259, 1652.9930, 1933.0086, and 2927.4561. The intact-mass profile supports the presence of detectable ions within the intended range but does not establish their chemical identities or peptide nature.

Table S1. Targeted mass spectrometry analysis of the relative abundance of 21 neurotransmitters in VBsV

No.	Metabolite	Normalized abundance ratio (%)	Category
1	5-Hydroxytryptamine	39.96	Monoamine neurotransmitter
2	L-Glutamic acid	32.21	Amino acid neurotransmitter
3	Histamine	19.88	Biogenic amine
4	3-Hydroxytyramine	7.04	Catecholamine
5	Tyramine	0.434	Trace amine
6	L-glutamine	0.294	Amino acid / glutamate precursor
7	L-tryptophan	0.116	Indole amino acid / precursor
8	L-tyrosine	0.034	Aromatic amino acid / catecholamine precursor
9	4-Aminobutyric acid	0.022	Amino acid neurotransmitter
10	Kynurenic acid	0.004	Tryptophan–kynurenine pathway
11	L-histidine	0.003	Amino acid / histamine precursor
12	Tryptamine	0.002	Indoleamine
13	L-Kynurenine	0.0008	Tryptophan–kynurenine pathway
14	Quinolinic acid	0.0006	Tryptophan–kynurenine pathway
15	Levodopa	0.0005	Catecholamine precursor
16	5-Hydroxyindole-3-acetic acid	ND	Serotonin metabolite
17	Melatonin	ND	Indoleamine
18	5-Hydroxytryptophan	ND	Serotonin precursor
19	Acetylcholine chloride	ND	Cholinergic neurotransmitter
20	DL-noradrenaline hydrochloride	ND	Catecholamine
21	DL-adrenaline hydrochloride	ND	Catecholamine

ND: Indicates no detection.

Table S2. Targeted mass spectrometry analysis of the relative abundance of 154 energy metabolites in VBsV.

No.	Metabolite	MS response (a.u.)	Metabolic class
1	Inosine	84,146,482	Purine nucleoside
2	L-Glutamate	33,844,330	Amino acid / neuroactive metabolite
3	L-Leucine (Leu)	21,609,445	Branched-chain amino acid
4	GABA	19,985,883	Amino acid neurotransmitter
5	Adenosine	16,504,650	Purine nucleoside
6	Guanosine	15,803,961	Purine nucleoside
7	L-Aspartic Acid (Asp)	14,898,264	Amino acid
8	L-Valine (Val)	13,666,965	Branched-chain amino acid
9	L-Proline (Pro)	8,385,992	Amino acid
10	Adenine	6,431,246	Purine base
11	Uridine	4,496,251	Pyrimidine nucleoside
12	Malate	4,090,708	TCA cycle intermediate
13	Hypoxanthine	3,983,261	Purine base
14	Fumarate	2,406,938	TCA cycle intermediate
15	Tau (Taurine)	2,376,417	Sulfur-containing amino acid derivative
16	L-Isoleucine (Ile)	1,844,505	Branched-chain amino acid
17	Cytosine	1,021,199	Pyrimidine base
18	Kynurenate	842,954	Tryptophan metabolite
19	L-Phenylalanine (Phe)	835,407	Aromatic amino acid
20	Citrate	736,156	TCA cycle intermediate
21	2'-Deoxyinosine	634,914	Purine deoxynucleoside
22	5-Oxoproline	507,519	Amino acid derivative
23	Creatinine	483,254	Creatine metabolism product
24	Guanine	409,756	Purine base
25	G1P	367,021	Sugar phosphate
26	Isocitric acid	356,764	TCA cycle intermediate
27	cAMP	253,658	Cyclic nucleotide
28	Beta-Ala (beta-Alanine)	240,694	Amino acid derivative
29	dT (Thymidine)	210,255	Pyrimidine deoxynucleoside
30	cGMP	177,837	Cyclic nucleotide

Table S2. Cont. Targeted mass spectrometry analysis of the relative abundance of 154 energy metabolites in VBsV.

No.	Metabolite	MS response (a.u.)	Metabolic class
31	L-Carnosine	161,722	Dipeptide
32	GSSG	87,797	Oxidized glutathione
33	SAM (S-Adenosylmethionine)	87,203	Methyl donor / sulfonium compound
34	L-1-Pyrroline-5-carboxylic acid	80,338	Proline/hydroxyproline metabolite
35	cis-aconitate	72,563	TCA cycle intermediate
36	Salicylic acid	65,429	Phenolic acid
37	GSH (Glutathione)	48,597	Reduced glutathione
38	Beta-Aminobutyric Acid	44,231	Amino acid derivative
39	acetyl-CoA	42,056	Acyl-CoA / acetyl donor
40	Thymine	41,010	Pyrimidine base
41	GMP (Guanosine monophosphate)	40,905	Purine nucleotide
42	Lactic acid	34,559	Glycolytic product
43	dGDP	33,377	Purine deoxynucleotide
44	Itaconate	29,499	TCA-derived immunometabolite
45	CoA (coenzyme A)	20,845	Coenzyme
46	Urate	19,512	Purine catabolite
47	NADP+	16,746	Redox cofactor
48	O-Acetyl-Ser	16,335	Amino acid derivative
49	Abscisic acid	11,861	Plant hormone-like small molecule
50	R5P (D-Ribose 5-phosphate)	11,804	Sugar phosphate
51	GDP	10,210	Purine nucleotide
52	ADP	9,560	Purine nucleotide
53	dCDP	9,422	Pyrimidine deoxynucleotide
54	N-Acetyl-L-Asp	8,921	Aspartate-derived metabolite
55	L-Argininosuccinic acid	8,706	Urea-cycle intermediate
56	UTP	5,995	Pyrimidine nucleotide
57-154	Others	Not detected	/
